# A Systematic Review and Meta-Analysis of Patient Preferences for Combination Thyroid Hormone Treatment for Hypothyroidism

**DOI:** 10.3389/fendo.2019.00477

**Published:** 2019-07-24

**Authors:** Amit Akirov, Rouhi Fazelzad, Shereen Ezzat, Lehana Thabane, Anna M. Sawka

**Affiliations:** ^1^Department of Endocrine Oncology, Princess Margaret Cancer Centre, Toronto, ON, Canada; ^2^Institute of Endocrinology, Beilinson Hospital, Petach Tikva, Israel; ^3^Sackler School of Medicine, Tel Aviv University, Tel Aviv, Israel; ^4^Princess Margaret Cancer Centre, University Health Network Library and Information Services, Toronto, ON, Canada; ^5^Department of Health Research Methods, Evidence, and Impact, McMaster University, Hamilton, ON, Canada; ^6^Division of Endocrinology, University Health Network and University of Toronto, Toronto, ON, Canada

**Keywords:** hypothyroidism, thyroid hormone, levothyroxine, triiodothyronine, systematic review, meta-analysis, randomized controlled trials

## Abstract

**Background:** The standard of care in management of hypothyroidism is treatment with levothyroxine (L-T4). Sometimes patients are dissatisfied with L-T4 and the combination of levo-triiodothyronine (L-T3) with L-T4 is considered.

**Methods:** We performed a systematic review and meta-analysis of blinded randomized controlled trials (RCTs), reporting how often hypothyroid patients prefer combination L-T3/L-T4 treatment to L-T4 alone. We also explored for explanatory factors for combination therapy preference in sensitivity analyses examining trial, patient, and disease characteristics. Potential dose-response relationships were explored using meta-regression analyses. We searched 9 electronic databases (from inception until February, 2019), supplemented with a hand-search. Two reviewers independently screened abstracts and citations and reviewed full-text papers, with consensus achieved on the included studies. Two reviewers independently critically appraised the quality of included studies and abstracted the data. Random effects meta-analyses were reported for the percentage of patients preferring combination L-T3/T-T4 therapy over L-T4 alone. A binomial distribution of choices (i.e., preference of combination therapy or no preference for combination therapy) was assumed.

**Results:** We included 7 blinded RCTs including 348 hypothyroid individuals in the primary meta-analysis. The pooled prevalence rate for preference of combination therapy over L-T4 was 46.2% (95% confidence interval 40.2%, 52.4%) (*p* = 0.231 for the difference from chance). There was no significant statistical heterogeneity among study results (Q = 7.32, degrees of freedom = 6, *p* = 0.293, *I*^2^ = 18.0%). In sensitivity analyses, combination treatment preference was explained in part by treatment effects on TSH concentration, mood and symptoms, but not quality of life nor body weight. In a secondary dose-response meta-regression analyses, a statistically significant association of treatment preference was identified for total daily L-T3 dose, but not L-T3:L-T4 dose ratio.

**Conclusions:** In conclusion, in RCTs in which patients and investigators were blinded to treatment allocation, approximately half of participants reported preferring combination L-T3 and L-T4 therapy compared to L-T4 alone; this finding was not distinguishable from chance. An observed potential positive L-T3 dose effect on treatment preference deserves further study, with careful consideration of thyroid biochemical indices and patient reported outcomes.

## Introduction

Internationally, levothyroxine (L-T4) treatment is the established first choice as a standard of care in the management of hypothyroidism (1–7). However, some patients are dissatisfied with L-T4 standard care treatment (8). Factors contributing to dissatisfaction with thyroid hormone treatment include persistent hypothyroid symptoms, such as excess weight, fatigue, mood problems, or memory/cognitive concerns (8). In the clinical context of persistent symptoms after achieving a normalized thyroid stimulating hormone (TSH) concentration on L-T4, after excluding or managing other potential causes of symptoms, patients and clinicians sometimes consider utilizing alternative thyroid hormone preparations, such as combination therapy using L-T4 and levo-triiodothyronine (L-T3). The rationale for this approach would be normalizing potentially low tissue T3 levels, which are not readily measurable. We conducted a systematic review and meta-analysis, examining how often hypothyroid patients prefer L-T3/L-T4 combination therapy over L-T4 monotherapy. In order to minimize the risk of bias, we restricted our review to blinded randomized controlled trials (RCTs). We also explored for explanatory factors relating to patient preferences.

## Methods

Our systematic review was registered (PROSPERO CRD42019123920). We included blinded RCTs, examining how often hypothyroid adult patients prefer combination L-T3/L-T4 therapy, compared to the standard of care of L-T4 monotherapy. Trial settings were restricted to be ambulatory outpatient clinics (i.e., not hospitalized patients) and participants were required to be aged ≥ 18 years of age, with hypothyroidism of any etiology. Trials were required to report some level of blinding, including blinding of study participants. All studies were required to have measured thyroid function in study participants using a thyroid stimulating hormone (TSH) measurement. Studies focusing on desiccated thyroid hormone were excluded. Due to limited resources for translation, only English language studies were included. For overlapping or duplicate studies reporting the same primary outcome, the largest study was included. As we expected strong reader interest in factors explaining patient preferences, we separately abstracted data from secondary explanatory analyses (such as deiodinase polymorphism status), which would typically be published in subsequent publications from the original studies. The explanatory data was not included in the meta-analysis of combination therapy preference rate.

An experienced library information specialist (RF) executed a comprehensive search strategy from inception to February 2019 in the following databases: MEDLINE, Ovid MEDLINE Epub Ahead of Print and In-Process and Other Non-Indexed Citations, Embase Classic + Embase, “Cochrane Central Register of Controlled Trials,” “Cochrane Database of Systematic Reviews,” Emcare, and PsycInfo all from the OvidSP platform; Web of Science from the Clarivate Analytic, and ClincalTrials.gov. We limited our search to adults (age ≥ 18 years) and the following types of studies: randomized controlled trials, systematic reviews, and meta-analyses. There was no language restriction on the search. Where available, both controlled vocabulary terms (“exploded” where applicable), and text words were used to identify as many relevant results as possible (Supplementary Data). We supplemented the electronic search by cross-referencing included papers, relevant sections of clinical practice guidelines, relevant systematic and narrative reviews, as well as reviewing the personal files of one of the authors who had participated in development of a hypothyroidism clinical practice guideline (AMS).

Two investigators (AA and AMS) independently, in duplicate, screened citations from the electronic search, reviewed full-text papers for inclusion, critically appraised the quality included studies, and abstracted the data. Consensus was achieved for inclusion of papers and abstracted data by discussion of reviewers; a third reviewer/clinical content expert (SE) was consulted in the event of any discrepancies that could not otherwise be resolved by reviewer discussion. We contacted the corresponding authors of original studies if there were questions relating to potential eligibility or results of studies or the results. The risk of bias of included trials was evaluated using the most current Risk of Bias evaluation tool developed by the Cochrane Collaboration (ROB 2.0) (9). The systematic review was reported according to PRISMA standards (10).

Descriptive data were summarized as numbers and percentages for categorical data and means or medians and standard deviations or ranges for continuous data. We performed random effects meta-analyses, estimating the percentage (with 95% confidence intervals, CI) of patients preferring combination L-T4 with L-T3 (any dose) over L-T4 monotherapy (Comprehensive Meta-Analysis software, version 2.0). A binomial distribution for preferring (or not preferring) combination therapy was assumed, such that a significant preference in combination therapy (beyond chance) would be defined by the lower limit of the 95% CI exceeding 50%. Individuals who preferred L-T4 or those who had no treatment preference, were judged to not prefer combination therapy. For the primary meta-analysis, treatment preference was evaluated only in individuals who had been exposed to L-T3 in a random fashion during the trial (i.e., individuals not receiving L-T3 at any point in the trial or those assigned L-T3 in non-random fashion, were not included). We evaluated for statistical heterogeneity in the meta-analysis using a Cochrane's Q (chi-squared) test (11) and an I^2^ estimate (12). We planned to evaluate for potential publication bias using a funnel plot (13), assuming at least 10 studies were included in the meta-analysis (for meaningful interpretation). We planned to explore for sources of heterogeneity of combination therapy treatment preference by performing the following sensitivity analyses (examining for difference in treatment effects among studies according to study characteristics): (a) study characteristics (study quality, study drug treatment duration [3 months or less, or longer than 3 months]), (b) participant characteristics (mean or median age <50 years or ≥ 50 years], sex [inclusion of any men], molecular characteristics, treatment (frequency of daily treatment dosing), other treatment effects (differential changes between groups on TSH concentration, quality of life, mood, psychological outcomes, or body weight), and disease characteristics (etiology of hypothyroidism, i.e., inclusion of any patients who had thyroidectomy or radioactive iodine treatment as an etiology for hypothyroidism). Fixed effects univariate meta-regression was performed to investigate for any dose-effect on thyroid hormone treatment preference, relating to study- and study subgroup- specific combination therapy dose, specifically: L-T3 total daily dose (ug) and L-T3:L-T4 ratio of daily dose. These variables were calculated for the hypothetical scenario of an individual receiving a baseline L-T4 dose of 100 ug daily, in order to account for differences in calculation of combination therapy dose among trials (i.e., dose ratios or fixed dose substitutions). We utilized the L-T3:L-T4 ratio (and not vice versa), to enable inclusion of data from individuals receiving a dosage of 0 ug of L-T3 (in the case of individuals randomized to an L-T4 arm, in a parallel design trial). Thus, in the case of parallel design randomized controlled trials, the L-T4 arm data (not exposed to combination therapy) was not included in the primary analysis of prevalence of combination therapy preference but would be eligible for inclusion in the secondary meta-regression dose analysis, where the outcome was study drug treatment preference (i.e., placebo compared to pre-trial L-T4 use in the case of parallel design trials or combination therapy compared to intra-trial L-T4 use for cross-over trials). We defined statistical significance of all analyses at an alpha level of 0.05; however, in examining for heterogeneity using Cochrane's Q test, we set that alpha level at 0.10 (11).

## Results

**Figure 1:**
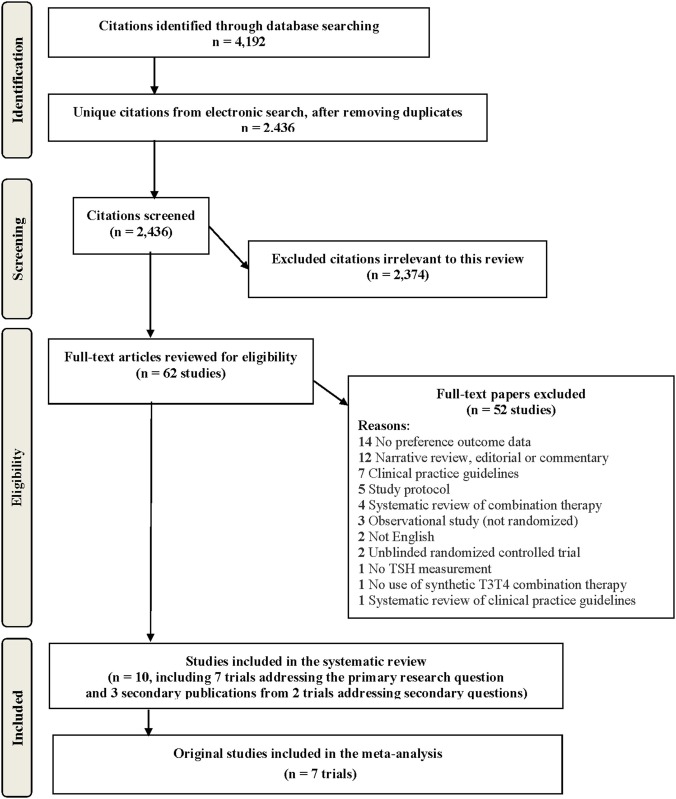
Study flow diagram.

As detailed in our study flow diagram (Figure 1), we retrieved a total of 4,192 citations from our electronic searches, ultimately yielding 2,436 unique citations after removing any duplicates. References from the hand search were all included in the electronic database searches, so the hand search yielded no additional relevant papers. We reviewed 62 full-text papers for eligibility and there were 7 trials included in the systematic review and meta-analysis (14–20). Relevant data on secondary subgroup analyses relating to molecular data were reported in two additional publications for the trial of Appelhof et al. [original publication (14), secondary publications (21, 22)] as well as Nygaard et al. [original publication (18), secondary publication (23)]. The reviewed full-text papers excluded from this review, and the reasons for exclusion are shown in the Supplementary Table 1. We excluded some RCTs comparing combination therapy compared to levothyroxine for the following reasons: (a) no data on patient preference—Clyde et al. (24), Kaminski et al. (25), Saravanan et al. (26), Sawka et al. (27), Siegmund et al. (28), Valizadeh et al. (29), (b) no blinding—Fadayev et al. (30), and (c) no TSH measurement (so safety and appropriateness of thyroid hormone dosing could not be established)—Smith et al. (31).

A summary of the details of the RCTs included in both the systematic review and meta-analysis is shown in Table 1. Of the included studies, five were conducted in Europe (14–18), one was conducted in the United States (19), and one was conducted in Australia (20). The number of participants randomized ranged from 13 to 141 hypothyroid patients (14–20). The majority of participants in the studies were female, with two studies recruiting only females (16, 17). Furthermore, the mean age of participants was younger than 50 years in all of the studies (14–20). The etiology of hypothyroidism was autoimmune primary hypothyroidism in the majority of participants in 5/7 studies (14, 17–20). None of the studies included patients with secondary hypothyroidism due to hypothalamic/pituitary disease. Of the 6 studies reporting on recruitment setting (14–16, 18–20), 5 reported recruiting participants in ambulatory Endocrinology clinics (15, 16, 18–20), and one reported recruiting participants from primary care practices (14). One study used a parallel design (14), whereas the other 6 studies utilized a cross-over design (15–20). Pre-trial L-T4 dose was required to be stable for at least 2 months in one study (20), 3 months in 2 studies (15, 19), 6 months in 2 studies (14, 18), and 1 year in one study (17); there was no reported requirement for duration of pre-trial stability of levothyroxine dosage in one study of surgically-treated patients who had Graves disease (16). The study treatment periods ranged from 5 to 15 weeks (14–20). The details of combination therapy are shown in Table 1; only one study (14) reported twice daily administration of L-T3. A summary of the risk of bias of included trials is shown in Table 2.

**Table 1:** Study and participant characteristics in the included randomized controlled trials.

**References**	**Country**	**Trial design**	Number randomized participant (Number reporting treatment preference outcome)^*^	**Females**	**Mean age (Standard deviation) (years)**	**Etiology hypothyroidism**	Description of combination L-T3^*^ and L-T4^†^ therapy	**Study funding and industry participation**
Appelhof et al. (14)	Netherlands	Parallel	141 (92 L-T3 arm, 48 L-T4 arm)	85% (120/141)	46.8–49.8 (9.4–9.8)	Autoimmune primary hypothyroidism (141/141, 100%)	Twice daily dosing. In respective study arms, 25 ug L-T4 removed and added L-T3, aiming for L-T4:L-T3 ratio of 5:1 or 10:1. For patient on 100 mcg LT4—for 5:1 becomes 75 ug L-T4 and 15 ug L-T3, for 10:1 becomes 75 ug L-T4:7.5 ug L-T3.	Academic institutional funding, Study medication provided by Merck, Netherlands
Bunevicius et al. (15)	Lithuania	Cross-over	35 (33)	94% (31/33)	46 (13)	Autoimmune primary hypothyroidism (16/33, 48%), thyroid cancer (17/33, 52%)	Once daily dosing. 12.5 ug L-T3 substituted for 50 ug of the usual L-T4 dose. For patient on 100 ug L-T4 becomes 50 ug L-T4 and 12.5 ug L-T3 (inferred 4:1).	Funding not reported. Study medication provided by Berlin-Chemie.
Bunevicius, et al. (16)	Lithuania	Cross-over	13 (10)	100% (13)	34 (NR^‡^)	Surgically-treated Graves disease (13/13, 100%)	Once daily dosing. 10 ug L-T3 substituted for 50 ug of the usual L-T4 dose. For patient on 100 ug L-T4, becomes 50 ug L-T4 and 10 ug L-T3 (inferred 5:1).	Not reported.
Escobar-Morreale et al. (17)	Spain	Cross-over	28 (26)	100% (28/28)	48 (11)	Autoimmune primary hypothyroidism (23/28, 82%); RAI-treated^μ^ Graves disease or Toxic Multinodular Goiter (5/28, 18%)	Once daily dosing. 5 ug L-T3 substituted for 25 ug of the usual L-T4 dose (all patients had baseline pre-trial L-T4 dose of 100 ug, so calculate 75 ug L-T4 and 5 ug L-T3, inferred ratio 15:1).	Academic and industry (Merck KgaA) funding.
Nygaard et al. (18)	Denmark	Cross-over	68 (59)	93% (55/59)	46.5−47.6 (12.3−13.1)	Autoimmune primary hypothyroidism (68/68, 100%)	Once daily dosing. 20 ug of L-T3 substituted for 50 ug of the usual L-T4 dose. For patient on 100 ug L-T4, becomes 50 ug L-T4 and 20 ug L-T3 (inferred 2.5:1).	Academic foundation funding.
Rodriguez et al. (19)	United States	Cross-over	30 (27)	83% (25/30)	47.5 (12.9)	Autoimmune primary hypothyroidism (23/30, 77%), Thyroidectomy (3/10, 10%), RAI-treated (4/30, 13%)	Frequency of daily dosing not reported (assume once a day). Aim for 5:1 ratio of L-T4:L-T3. 10 ug L-T3 substituted for 50 ug of the usual L-T4 dose. For patient on 100 ug L-T4, becomes 50 ug L-T4 and 10 ug L-T3.	Academic funding from the National Institutes of Health. Medication provided by King Pharmaceuticals.
Walsh et al. (20)	Australia	Cross-over	110 (101)	92% (101/110)	47.7 (11.7)	Autoimmune primary hypothyroidism (94/110, 85%) thyroidectomy for non-malignant reason (12/110, 11%), RAI (4/110, 4%).	Once daily dosing. 10 ug L-T3 substituted for 50 ug of the usual L-T4 dose. For patient on 100 ug L-T4, becomes 50 ug L-T4 and 10 ug L-T3 (inferred 5:1).	Academic institutional funding, L-T3 donated by Boots, Australia

* *L-T3, levo-triiodothyronine*.

† *L-T4, levothyroxine*.

‡ *NR, not reported*.

μ *RAI-treated, radioactive iodine-treated*.

**Table 2:** Quality assessment of the included randomized controlled trials.

**Reference**	**Selection Bias (Randomization and allocation concealment)**	**Risk of bias due to deviation from intended interventions (e.g., adherence)**	**Risk of bias due to missing outcome data**	**Risk of bias in measurement of levo-triiodothyronine (L-T3) preference outcome with explanation**	**Selective outcome reporting**
Appelhof et al. (14)	Low	Some concerns^*^	Low	Some concerns^μ^ Explanation: Subjective appreciation of L-T3 compared to pre-trial L-T4 was rated by the participants on a 5-point scale (much better, somewhat better, the same, somewhat worse, or much worse), and those who indicated much or somewhat better were categorized as preferring L-T3. Unclear if validated scale.	Low
Bunevicius et al. (15)	Some concerns^*^	Some concerns^*^	Low	Some concerns^μ^ Explanation: At the end of the trial, participants asked which treatment was preferred. Unclear if standardized instrument or wording or if what response options may have been provided.	Low
Bunevicius et al. (16)	Some concerns^*^	Some concerns^*^	High (23% loss randomized participants)	Some concerns^μ^ Explanation: At the end of the trial, participants asked which treatment was preferred. Unclear if standardized instrument or wording or if what response options may have been provided.	Low
Escobar-Morreale et al. (17)	Low	Low	Low	Some concerns^μ^ Explanation: At the end of the trial, participants asked which treatment was preferred. Unclear if standardized instrument or wording or if what response options may have been provided.	Low
Nygaard et al. (18)	Some concerns^†^	Some concerns^*^	Some concerns (13% loss randomized participants)	Some concerns^μ^ Explanation: At the end of the trial, participants asked which treatment was preferred. Unclear if standardized instrument or wording or if what response options may have been provided.	Low
Rodriguez et al. (19)	Low	Some concerns^*^	Some concerns (10% loss randomized participants)	Some concerns^μ^ Explanation: At the end of the trial, participants asked which treatment was preferred. Unclear if standardized instrument or wording or if what response options may have been provided.	Low
Walsh et al. (20)	Some concerns^‡^	Low	Low	Some concerns^μ^ Explanation: At the end of the trial, participants asked which treatment was preferred. Unclear if standardized instrument or wording or if what response options may have been provided.	Low

* *Insufficient detail reported in the manuscript*.

† *The levothyroxine component of combination therapy was open label for dose adjustment*.

‡ *Sealed envelopes were used but there was no report of whether these were opaque (to ensure that the treatment allocation was not visible through the envelope)*.

μ *Some concerns, if there was no validated questionnaire outcome measure for treatment preference*.

**Figure 2:**
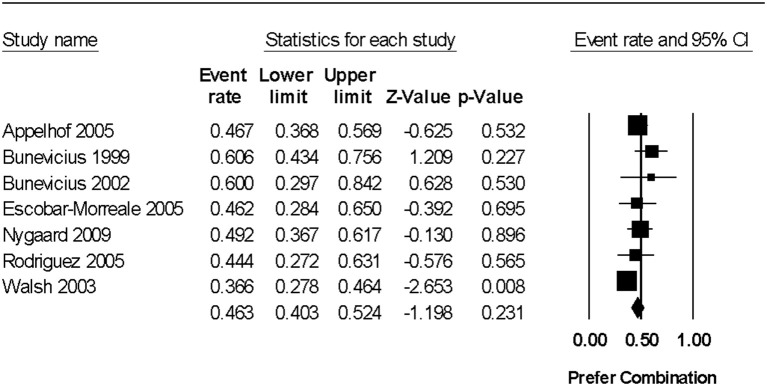
Forest plot from a random effects meta-analysis examining prevalence of preference of combination levo-triodothyronine (L-T3) and levothyroxine (L-T4) therapy over L-T4 alone. 95% CI, 95% confidence interval. For the studies reporting the number of individuals who had no preference (and thus were assumed to favor standard care), the rates were as follows: Bunevicius et al. (15)−33.3% (11/33), Bunevicius et al. (16)−20.0% (2/10), Nygaard et al. (18)−35.6% (21/59), Rodriguez et al. (19)−29.6% (8/27), and Walsh et al. (20)−17.8% (18/101). It is not known if the individuals with no preference were indifferent or indecisive (i.e., unable to make a decision).

In a random effects meta-analysis, the pooled prevalence rate for preference of combination therapy over L-T4 was 46.2% (95% CI 40.2%, 52.4%) (*p* = 0.231 for the difference from chance, using data from 7 trials including 348 hypothyroid individuals) (Figure 2). There was no significant statistical heterogeneity among study results (*Q* = 7.32, degrees of freedom [*df* ] = 6, *p* = 0.293, *I*^2^ = 18.0%). A funnel plot investigating for publication bias was not performed due to an insufficient number of studies for meaningful interpretation (i.e., fewer than 10 trials in the meta-analysis).

In spite of the lack of statistically significant heterogeneity in our primary meta-analysis, we proceeded with planned sensitivity analyses, to explore for any potential differences in treatment benefits according to patient, study, and disease characteristics. We were not able to examine the impact of study duration as all of the included trials (14–20) were ≤ 3 months in duration; furthermore, we were not able to examine any potential impact of age, as the mean age of study participants was relatively young (<50 years of age) in all trials (14–20). In terms of study quality, there was no significant difference in combination therapy preference between 4 studies in which there were some concerns about selection bias (randomization/concealment of allocation) (15, 16, 18, 20) compared to 3 studies which were considered at low risk of bias for that variable (14, 17, 19) (between study heterogeneity *Q* = 0.027, *df* = 1, *p* = 0.871). There was also no significant difference between 5 studies that included some men (14, 15, 18–20), compared to two that included only women (16, 17) (*Q* = 0.196, *df* = 1, *p* = 0.658). Furthermore, there were no significant difference between 5 trials that included individuals who had a thyroidectomy or radioactive iodine treatment (15–17, 19, 20) compared to 2 trials that included only individuals with autoimmune primary hypothyroidism (14, 18) (*Q* = 0.083, *df* = 1, *p* = 0.773). In examining the effect of frequency of dosing of combination therapy, there was no significant difference between one study that utilized twice daily dosing (14) compared to the 6 other studies that utilized once daily dosing or did not report on dosing (assuming once daily dose) (15–20) (*Q* = 0, *df* = 1, *p* = 0.998). In examining whether trials with end of trial TSH differences between treatment groups were associated with differences in treatment preferences, a trend for a possible marginal association was observed. Specifically the preference rate for combination therapy was 38.6% (95% CI 30.5%, 47.4%) in the 2 studies where the TSH was significantly higher in the combination therapy group compared to the L-T4 group (17, 20), 46.7% (95% CI 36.8%, 56.9%) in one trial where TSH was reduced in the combination therapy group compared to the L-T4 group (14) and 51.9% (95% CI 43.2%, 60.4%) in the 4 trials where end of trial TSH was not significantly different between treatment groups (15, 16, 18, 19) (between group difference for categories of TSH differences, *Q* = 4.504, *df* = 2, *p* = 0.105). In summary, study quality (reflected by randomization/concealment of allocation method), inclusion of males, inclusion of individuals who had a thyroidectomy or radioactive iodine treatment, and frequency of combination therapy daily dosing, did not explain combination therapy preference; however a possible marginal relationship between end of study TSH differences and treatment preference was observed.

**Figure 3:**
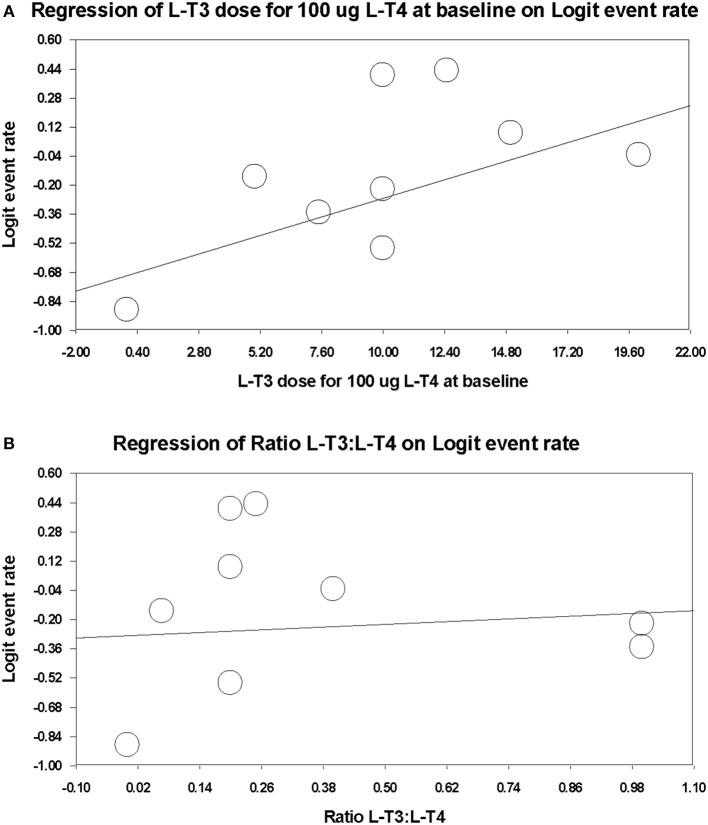
Meta-regression plots examining for any dose-response relationship between combination therapy dose and treatment preference over L-T4 monotherapy (L-T4 monotherapy during or before trial). **(A)** Total daily L-T3 dosage (ug) on combination therapy. **(B)** Ratio of L-T3 divided by L-T4 dosage (ug) on combination therapy.

In order to investigate any relationship between dose and treatment preference over L-T4 monotherapy taken during or before the trial, the respective L-T3 and L-T4 total daily dosage, and the ratio of these two doses, was calculated for a hypothetical baseline L-T4 dosage of 100 ug/day, according to each respective trial protocol (Table 2). Respective fixed effects meta-regression analyses were performed (Figure 3). Data from the L-T4 monotherapy arm in the parallel design randomized trial of Appelhof et al. (14) was included in these analyses, assuming that the L-T4 baseline dosage was continued and the L-T3 dosage was 0 ug/day. Data from 7 trials of 396 participants [incorporating 3 subgroups from the trial by Appelhof et al. (14)] were used in the meta-regression analyses. There was a statistically significant positive association between total daily L-T3 dosage (which ranged from 0 to 20 ug/day) and treatment preference (slope regression model 0.043, 95% CI 0.007, 0.078, *p* = 0.020) (Figure 3A). However, there was no significant association of treatment preference with the L-T4:L-T3 ratio of 1:0 to 4:1) (slope 0.124, −0.489, 0.738, *p* = 0.691) (Figure 3B).

We performed several sensitivity analyses of combination therapy preferences, where we grouped trials according to changes in other specific outcomes. Specifically, grouping studies that demonstrated differences between treatment groups for validated measures of quality of life, changes in body weight, mood, and symptoms. We found no significant difference in rate of preference for combination therapy in comparing one trial reporting improved quality of life with combination therapy (18) to 3 trials where there was no significant treatment group difference in any quality of life measure (14, 17, 20) (*Q* = 1.644, *df* = 2, *p* = 0.439). Furthermore, there was no significant difference in treatment preference rate in comparing one study in which body weight was statistically significantly reduced in the combination therapy group (14) to 5 other trials reporting no significant body weight difference (*Q* = 0.637, *df* = 1, *p* = 0.723). However, a marginally higher rate of preference for combination therapy (53.2%, 95% CI 42.9%, 63.2%) was observed in two trials reporting significant improvement in mood and symptoms (respectively) with combination treatment (15, 18), compared to 5 other trials where there was no significant difference between treatment groups in either measure (43.1%, 95% CI 37.1%, 49.2%) (14, 16–20) (between group difference *Q* = 2.762, *df* = 1, *p* = 0.097). In summary, the minority of trials reporting improvement in mood and symptoms, tended to report higher rates of combination therapy preference.

Although none of the primary reports of the trials in this systematic review included molecular biomarker data, given the importance of potential relationship between molecular characteristics of patients and treatment preference, reports of secondary publications from included trials were descriptively summarized. The methodologic quality of respective secondary analysis papers (21–23) was considered consistent with that of the original trials, so is not reported separately. In secondary analyses of original randomized trial data (14), Appelhof et al. compared rates of preference for combination therapy, according to genetic polymorphism status of type 2 deiodinase enzymes for Thr92Ala and ORFa-Gly3Asp (also known as rs12885300) (21). The prevalence rate of the Thr92Ala polymorphism among 141 trial participants was as follows: 74 (52%) heterozygous, 20 (14%) homozygous, and 47 (33%) wild type (21). The number of individuals and percentage with the ORFa-Gly3Asp polymorphism was: 52 (37%) heterozygous, 19 (13%) homozygous, and 70 (50%) wild type (21). Among the 92 patients who received combination therapy, no significant differences in rates of combination therapy preference were observed according to Thr92Ala polymorphism genotype (53% heterozygous, 39% homozygous, 41% wild type) nor ORFa-Gly3Asp genotype (49% heterozygous, 43% homozygous, and 46% wild type) (21). The authors concluded there was no association between D2 polymorphisms and well-being or subjective preference for combination treatment over L-T4 monotherapy (21). In another secondary analysis of the same original trial by Appelhof et al. (14), Van der Deure et al. (22) examined polymorphisms in OATP1C1 gene, encoding a protein capable of thyroid hormone transport into the brain. Genotyping was successfully executed in140/141 patients in the original trial (22). The prevalence of OATP1C1-intron3C>T, OATP1C1-Thr143 and OATP1C1-3035T alleles were 46, 3, and 43%, respectively (22). Among 92 trial patients who received combination therapy, there was no significant difference in combination therapy preference according to genotype status: nOATP1C1-intron 3C (*p* = 0.68); OATP1C1-pro143Thr (*p* = 0.22), or OATP1C1-C3035T (*p* = 0.95 for respective chi-squared tests). However, in a secondary analysis of the trial from Nygaard et al. (18), Carlé et al. reported that in a subgroup of 45 patients from the 59 participants completing the original trial (18), the presence of the combination of 2 polymorphisms (rs225014 encoding the DIO2 enzyme and rs12885300 encoding the MCT10 transporter) was associated with a higher rate of preference for combination treatment: 63% if one polymorphism present, 100% if both polymorphisms present, and 42% if wild type (*p* = 0.009) (23).

## Discussion

In conclusion, in this systematic review and meta-analysis of relatively short-term blinded RCTs, approximately 46% of adult hypothyroid patients preferred combination therapy with L-T3 and L-T4 and L-T3 over L-T4 monotherapy; yet these findings were not distinguishable from chance. Some differences between this study with prior guideline narrative summaries of combination therapy preference rates (1, 32), is our strict inclusion criteria relating to blinding (to minimize the risk of bias), exclusion data from any add-on non-randomized treatment arms (also to minimize the risk of bias), and the a priori definition of statistical significance relative to chance in our meta-analysis. The fundamental clinical assumption of our analyses was that patients either prefer combination therapy to the standard of care or not, so patients who prefer L-T4 monotherapy or those who have no preference, would be grouped together as they would be treated with the same standard of care of L-T4 monotherapy. We found no patient demographic, disease, or study characteristics associated with variability in combination therapy preference. Studies reporting improvements in symptoms and mood (15, 18), tended to report higher rates of preference rates for combination therapy. The types of physical and emotional symptoms that were reported on questionnaires to be improved in these studies (15, 18) included: feeling cold (15), blurred vision (15), nausea (15), fatigue (15), depression/sadness (15, 18), anger (15), confusion (15), fearfulness (15), irritability (15), anxiety (18), and general health (18). However, the majority of studies did not report any significant difference in quality of life (using validated quality of life questionnaires) nor body weight with combination therapy and patient preference did not vary with these measures. Significant weight loss was reported only in a high dose combination therapy arm (5:1 L-T4 to L-T3 dose ratio) in one trial, where the TSH was suppressed with combination therapy (14). There was some preference variability of marginal statistical significance associated with end of trial TSH difference between study groups; specifically, for trials reporting an end of trial combination therapy group TSH that was either significantly lower or higher than the L-T4 monotherapy arm, tended to be associated with diminished patient preference for combination therapy. A positive association of L-T3 total daily dosage and treatment preference was observed in an exploratory univariate meta-regression analysis, where the dosages of L-T3 varied from zero to 20 ug/day. We were not able to make any firm conclusion on any potential relationship between patient molecular characteristics and combination therapy due to paucity of data. The secondary analyses summarized in this review should be interpreted as hypothesis generating and further confirmatory research is needed.

It is important to acknowledge that in the clinical practice setting, patient preference rates for combination therapy may differ from those observed in blinded randomized trials, particularly if patients may have some negative pre-established perceptions of L-T4 therapy (nocebo effect) and positive expectations with combination therapy (particularly L-T3). In clinical practice, experiences of patients treated with combination therapy may be highly variable, including the reasons preferring combination therapy (or not), and the degree of benefits on symptoms, well-being, and functional ability. However, the potential therapeutic benefits are likely be enhanced in a supportive, encouraging clinical care environment where patients' symptoms and concerns are acknowledged and their views incorporated in medical decision-making. Of note, intense monitoring for treatment benefits (e.g., using detailed questionnaires) and adverse effects is expected to be more rigorous in a research trial setting compared to clinical practice. The experience of clinicians prescribing and adjusting the dose of combination therapy may be also different in clinical practice compared to the clinical trial setting (e.g., specialized clinical trial centers with investigators experienced in use of combination therapy). In an effort to address physician expertise, authors from the European Thyroid Association have recommended that only specialists accredited in Endocrinology or Internal Medicine should be the ones prescribing combination therapy (32). Yet even among endocrinologists, experience prescribing combination therapy may be variable. Some practical potential challenges in clinically utilizing combination therapy using commercially-available existing short-acting L-T3 preparations may include: complexity of administration of two different thyroid hormone preparations (often more than once a day for higher total daily doses), increased medication expense (the extent of which varies globally), and greater complexity/expense in medication monitoring (e.g., inclusion of T3 measurements in bloodwork, potentially including peak levels). Saravanan et al. have reported that in hypothyroid patients receiving combination therapy, where the baseline L-T4 dose is reduced by 50 ug and replaced with 10 ug of L-T3, peak blood free T3 levels rise 42% (4 h after dose administration) (33). As such, use of higher dose L-T3 in combination treatment may necessitate measurement of peak T3 blood levels and splitting of the doses of this hormone. The extent to which such levels are faithfully reflected by diverse tissue requirements further confounds optimal dosing of L-T4/L-T3. Authors from the European Thyroid Association have also suggested a relatively physiologic combination therapy dose ratio L-T4/L-T3 ranging from 13:1 to 20:1 by weight (administering L-T4 once daily and dividing the total daily L-T3 dose in two doses) (32). The ETA guidelines regarding combination therapy were developed to enhance safety (potentially due to harms from treatment with supraphysiologic doses of thyroid hormones) and to counter its indiscriminate use (32). The ETA guidelines have indicated that “the goal of L-T4/L-T3 combination therapy is to resolve persistent complaints despite a normal TSH in L-T4-treated hypothyroid patients” (32). Furthermore, in the interest of safety, the ETA guidelines have recommended close specialist follow-up, with dose adjustments intended to meet the goals of treatment (32). Authors from the Italian Society of Endocrinology and the Italian Thyroid Association have suggested a dose ratio of L-T4:L-T3 of 10 to 20:1, administered in divided daily doses (3). However, the suggested relatively physiologic L-T3 dosages are below that used in many of the trials included in this review. One of the potential risks of using higher dose L-T3 may be TSH suppression, particularly if the L-T4 dosage is not sufficiently reduced, and TSH suppression with combination therapy was reported in some of the included trials. The Italian guidelines also highlight the importance of close monitoring for potential adverse effects, including cardiovascular complications and osteoporosis (3). Relative contraindications to L-T3 combination therapy are important considerations. Clinical practice guideline authors sponsored various organizations have recommended avoiding combination L-T3/L-T4 therapy in the following groups: pregnant women (3, 6, 32, 34) the elderly (3) patients with known cardiac arrhythmias (6, 32), individuals with cardiac risk factors (3), patients with differentiated thyroid cancer with a high risk of disease progression or intermediate to high risk of adverse effects (3).

There are multiple strengths and several limitations of this systematic review and meta-analysis. An important strength is the systematic search for relevant citations conducted in multiple electronic databases by an experienced library information specialist (which was supplemented by a hand search). Furthermore, two reviewers independently review of citations and full-text papers in duplicate, with resolution of any discrepancies in inclusion of papers resolved in discussion with a third content expert reviewer. Two investigators also independently critically appraised included studies and abstracted the data, with the final consensus of reported results. We also contacted some authors of primary studies to obtain critically information, relating to study inclusion and results. Some limitations of this research include: exclusion of non-English studies (due to lack of resources for translation), lack of a comprehensive search of the gray literature, inclusion of a relatively small number of trials (such that publication bias could not be reliably assessed), some methodologic limitations of included trials, and short duration of included trials (precluding analysis of durability of patient preference over time). Additional potential limitations relating to treatment of hypothyroidism that were not addressed by this review nor the included studies include the potential for a symptom-optimized TSH goal that may be narrower than the traditional 95% reference range (35), drug interference with TSH secretion (36), management of treatment-refractory hypothyroidism (i.e., individuals requiring unusually high doses of thyroid hormones) (37), and consideration of interference with gastrointestinal absorption of thyroid hormones (38).

In conclusion, although L-T4 monotherapy is the standard of care in management of hypothyroidism in adults, dissatisfaction among some patients treated with L-T4 as well as significant uncertainties relating to thyroid hormone alternatives, highlights the critical need for more research on effective treatments to optimize the well-being and treatment satisfaction in this population.

## Data Availability

The raw data supporting the conclusions of this manuscript will be made available by the authors, without undue reservation, to any qualified researcher.

## Author Contributions

All of the authors provided input on the design of the study and reviewed the manuscript. RF conducted the electronic database search. AA and AS screened citations, reviewed the full-text papers, critically appraised included studies, abstracted the data, and drafted the manuscript. SE provided input, in the event consensus was not achieved by AA and AS on inclusion of studies. AS conducted the statistical analyses and the statistical methods and data were reviewed by LT.

### Conflict of Interest Statement

AS was a co-author of a clinical practice guideline on hypothyroidism (American Thyroid Association). The remaining authors declare that the research was conducted in the absence of any commercial or financial relationships that could be construed as a potential conflict of interest.
