# Supplementary material for: A Systematic Review and Meta-Analysis of Patient Preferences for Combination Thyroid Hormone Treatment for Hypothyroidism
Source: Front Endocrinol (Lausanne). 2019 Jul 24;10:477. doi: 10.3389/fendo.2019.00477 (PMC6667836; doi:10.3389/fendo.2019.00477)
Supplement: Supplementary file 1 [file Data_Sheet_1.PDF]

Supplementary Material

Supplementary Data. Electronic Search Strategy

A. Ovid MEDLINE(R) 1946 to February 08, 2019

#	Searches	Results	Type
1	Hypothyroidism/	26432	Advanced
2	hypothyr*.mp,kw.	39457	Advanced
3	hypo-thyr*.mp,kw.	60	Advanced
4	(thyroid? adj3 defic*).mp,kw.	1359	Advanced
5	(thyroid? adj3 insuffic*).mp,kw.	425	Advanced
6	(thyroid? adj3 failure?).mp,kw.	472	Advanced
7	(thyroid? adj3 low adj3 product*).mp,kw.	6	Advanced
8	(thyroid? adj3 under adj3 product*).mp,kw.	4	Advanced
9	(thyroid? adj3 underactive*).mp,kw.	19	Advanced
10	(tsh adj3 defic*).mp,kw.	357	Advanced
11	or/1-10	40565	Advanced
12	Triiodothyronine/	25009	Advanced
13	triiodothyronin*.mp,kw.	29852	Advanced
14	triiodo l thyronin*.mp,kw.	884	Advanced
15	triiodo-thyronin*.mp,kw.	55	Advanced
16	levotriiodothyronin*.mp,kw.	13	Advanced
17	liothyronin*.mp,kw.	199	Advanced
18	lio-thyronin*.mp,kw.	0	Advanced
19	liothyron*.mp,kw.	199	Advanced
20	lyothyronin*.mp,kw.	6	Advanced
21	levoliothyronin*.mp,kw.	0	Advanced
22	L-triiod?thyronin*.mp,kw.	696	Advanced
23	L-T3.mp,kw.	364	Advanced
24	LT3.mp,kw.	84	Advanced
25	6138-47-2.rn.	0	Advanced
26	6893-02-3.rn.	0	Advanced
27	or/12-26	30115	Advanced
28	Thyroxine/	36353	Advanced
29	levothyrox*.mp,kw.	2799	Advanced

30	laevothyrox*.mp,kw.	7	Advanced
31	levo-thyrox*.mp,kw.	75	Advanced
32	laevo-thyrox*.mp,kw.	2	Advanced
33	l-thyrox*.mp,kw.	3031	Advanced
34	levo-t.mp,kw.	2	Advanced
35	oroxin*.mp,kw.	15	Advanced
36	thyroxin*.mp,kw.	45563	Advanced
37	L-T4.mp,kw.	894	Advanced
38	LT4.mp,kw.	402	Advanced
39	51-48-9.rn.	0	Advanced
40	or/28-39	46566	Advanced
41	27 or 40	58100	Advanced
42	11 and 41	15167	Advanced
43	exp animals/ not (exp animals/ and exp humans/)	4545687	Advanced
44	42 not 43	11366	Advanced
45	limit 44 to "all child (0 to 18 years)"	3782	Advanced
46	limit 44 to "all adult (19 plus years)"	6314	Advanced
47	45 not 46	2354	Advanced
48	44 not 47	9012	Advanced
49	review/	2318258	Advanced
50	(medline or medlars or pubmed or grateful med or CINAHL or scisearch or psychinfo or psycinfo or psychlit or psyclit or handsearch* or hand search* or manual* search* or electronic database* or bibliographic database* or embase or lilacs or scopus or web of science).mp,kw.	161368	Advanced
51	49 and 50	117313	Advanced
52	"systematic review"/	101745	Advanced
53	systematic reviews as topic/	1982	Advanced
54	"scientific integrity review"/	246	Advanced
55	meta-analysis/	97015	Advanced
56	exp meta-analysis as topic/	17334	Advanced
57	exp randomized controlled trial/	476147	Advanced
58	exp randomized controlled trials as topic/	123352	Advanced
59	single-blind method/	26248	Advanced
60	double-blind method/	149490	Advanced
61	placebos/	34221	Advanced
62	"systematic review".pt.	101745	Advanced
63	meta-analysis.pt.	97015	Advanced

64	randomized controlled trial.pt.	475681	Advanced
65	meta-anal*.mp,kw.	142218	Advanced
66	metaanal*.mp,kw.	2173	Advanced
67	metanal*.mp,kw.	413	Advanced
68	(systematic* adj4 (review? or overview? or study or studies)).mp,kw.	146991	Advanced
69	cochrane database*.jn.	13620	Advanced
70	random*.mp,kw.	1097474	Advanced
71	quasirandom*.mp,kw.	94	Advanced
72	(pragmatic* adj4 (trial? or study or studies)).mp,kw.	3819	Advanced
73	(conceal* adj2 allocat*).mp,kw.	2255	Advanced
74	"single blind*".mp,kw.	31610	Advanced
75	"double blind*".mp,kw.	176707	Advanced
76	"triple-blind*".mp,kw.	531	Advanced
77	"single masked*".mp,kw.	323	Advanced
78	"double masked".mp,kw.	2851	Advanced
79	"triple masked".mp,kw.	47	Advanced
80	placebo*.mp,kw.	197050	Advanced
81	or/51-80	1357494	Advanced
82	48 and 81	562	Advanced
83	limit 48 to (meta analysis or randomized controlled trial or "systematic review" or systematic reviews as topic)	297	Advanced
84	82 or 83	562	Advanced

B. **Ovid MEDLINE(R) Epub Ahead of Print and In-Process & Other Non-Indexed Citations February 11, 2019**

#	Searches	Results	Type
1	hypothyr*.mp,kw.	3282	Advanced
2	hypo-thyr*.mp,kw.	10	Advanced
3	(thyroid? adj3 defic*).mp,kw.	133	Advanced
4	(thyroid? adj3 insuffic*).mp,kw.	38	Advanced
5	(thyroid? adj3 failure?).mp,kw.	42	Advanced
6	(thyroid? adj3 low adj3 product*).mp,kw.	0	Advanced
7	(thyroid? adj3 under adj3 product*).mp,kw.	0	Advanced
8	(thyroid? adj3 underactive*).mp,kw.	1	Advanced

9	(tsh adj3 defic*).mp,kw.	20	Advanced
10	or/1-9	3404	Advanced
11	triiodothyronin*.mp,kw.	875	Advanced
12	triiodo l thyronin*.mp,kw.	54	Advanced
13	triiodo-thyronin*.mp,kw.	1	Advanced
14	levotriiodothyronin*.mp,kw.	2	Advanced
15	liothyronin*.mp,kw.	45	Advanced
16	lio-thyronin*.mp,kw.	0	Advanced
17	liothyron*.mp,kw.	45	Advanced
18	lyothyronin*.mp,kw.	1	Advanced
19	levoliothyronin*.mp,kw.	0	Advanced
20	L-triiod?thyronin*.mp,kw.	14	Advanced
21	L-T3.mp,kw.	23	Advanced
22	LT3.mp,kw.	11	Advanced
23	levothyrox*.mp,kw.	591	Advanced
24	laevothyrox*.mp,kw.	1	Advanced
25	levo-thyrox*.mp,kw.	6	Advanced
26	laevo-thyrox*.mp,kw.	0	Advanced
27	l-thyrox*.mp,kw.	196	Advanced
28	levo-t.mp,kw.	0	Advanced
29	oroxin*.mp,kw.	5	Advanced
30	thyroxin*.mp,kw.	1801	Advanced
31	L-T4.mp,kw.	91	Advanced
32	LT4.mp,kw.	83	Advanced
33	or/11-32	2596	Advanced
34	10 and 33	1025	Advanced
35	"systematic review".pt.	9	Advanced
36	meta-analysis.pt.	42	Advanced
37	randomized controlled trial.pt.	277	Advanced
38	(systematic* adj4 (review? or overview? or study or studies)).mp,kw.	39527	Advanced
39	meta-anal*.mp,kw.	28289	Advanced
40	metaanal*.mp,kw.	364	Advanced
41	metanal*.mp,kw.	77	Advanced
42	random*.mp,kw.	152360	Advanced
43	quasirandom*.mp,kw.	56	Advanced
44	(pragmatic* adj4 (trial? or study or studies)).mp,kw.	889	Advanced
45	"single blind*".mp,kw.	2152	Advanced
46	"double blind*".mp,kw.	12302	Advanced

47	"triple-blind*".mp,kw.	201	Advanced
48	"single masked*".mp,kw.	40	Advanced
49	"double masked".mp,kw.	239	Advanced
50	"triple masked".mp,kw.	9	Advanced
51	placebo*.mp,kw.	18731	Advanced
52	or/35-51	196533	Advanced
53	34 and 52	107	Advanced

C. **Embase Classic+Embase 1947 to 2019 February 08**

#	Searches	Results	Type
1	hypothyroidism/	61071	Advanced
2	hypothyr*.mp,kw.	78362	Advanced
3	hypo-thyr*.mp,kw.	179	Advanced
4	(thyroid? adj3 defic*).mp,kw.	2325	Advanced
5	(thyroid? adj3 insuffic*).mp,kw.	820	Advanced
6	(thyroid? adj3 failure?).mp,kw.	830	Advanced
7	(thyroid? adj3 low adj3 product*).mp,kw.	15	Advanced
8	(thyroid? adj3 under adj3 product*).mp,kw.	5	Advanced
9	(thyroid? adj3 underactive*).mp,kw.	38	Advanced
10	(tsh adj3 defic*).mp,kw.	625	Advanced
11	or/1-10	80274	Advanced
12	liothyronine/	40555	Advanced
13	triiodothyronin*.mp,kw.	21777	Advanced
14	triiodo l thyronin*.mp,kw.	1040	Advanced
15	triiodo-thyronin*.mp,kw.	112	Advanced
16	levotriiodothyronin*.mp,kw.	17	Advanced
17	liothyronin*.mp,kw.	44357	Advanced
18	lio-thyronin*.mp,kw.	1	Advanced
19	liothyron*.mp,kw.	44357	Advanced
20	lyothyronin*.mp,kw.	6	Advanced
21	levoliothyronin*.mp,kw.	0	Advanced
22	L-triiod?thyronin*.mp,kw.	1025	Advanced
23	L-T3.mp,kw.	519	Advanced
24	LT3.mp,kw.	154	Advanced
25	6138-47-2.rn.	37904	Advanced
26	6893-02-3.rn.	37904	Advanced
27	or/12-26	48414	Advanced

28	thyroxine/	60507	Advanced
29	levothyrox*.mp,kw.	20623	Advanced
30	laevothyrox*.mp,kw.	14	Advanced
31	levo-thyrox*.mp,kw.	138	Advanced
32	laevo-thyrox*.mp,kw.	5	Advanced
33	l-thyrox*.mp,kw.	4794	Advanced
34	levo-t.mp,kw.	17	Advanced
35	oroxin*.mp,kw.	56	Advanced
36	thyroxin*.mp,kw.	76437	Advanced
37	L-T4.mp,kw.	1551	Advanced
38	LT4.mp,kw.	925	Advanced
39	51-48-9.rn.	15295	Advanced
40	or/28-39	89514	Advanced
41	27 or 40	106414	Advanced
42	11 and 41	30936	Advanced
43	(exp animals/ or exp animal experimentation/ or nonhuman/) not ((exp animals/ or exp animal experimentation/ or nonhuman/) and exp human/)	6906404	Advanced
44	42 not 43	25858	Advanced
45	limit 44 to (embryo <first trimester> or infant <to one year> or child <unspecified age> or preschool child <1 to 6 years> or school child <7 to 12 years> or adolescent <13 to 17 years>)	4311	Advanced
46	limit 44 to (adult <18 to 64 years> or aged <65+ years>)	11256	Advanced
47	45 not 46	3022	Advanced
48	44 not 47	22836	Advanced
49	"systematic review"/	193448	Advanced
50	"systematic review (topic)"/	23604	Advanced
51	exp meta analysis/	157696	Advanced
52	exp "meta analysis (topic)"/	39837	Advanced
53	network meta-analysis/	1527	Advanced
54	metaanaly*.mp,kw.	9756	Advanced
55	meta-analy*.mp,kw.	253782	Advanced
56	metanaly*.mp,kw.	656	Advanced
57	(systematic* adj3 (review? or study or studies or overview?)).mp,kw.	296799	Advanced
58	exp "randomized controlled trial (topic)"/	157829	Advanced
59	randomized controlled trial/	537934	Advanced
60	exp randomization/	81633	Advanced

61	placebo/	340941	Advanced
62	double blind procedure/	160578	Advanced
63	single blind procedure/	33932	Advanced
64	random*.mp,kw.	1602882	Advanced
65	quasirandom*.mp,kw.	184	Advanced
66	(pragmatic* adj4 (trial? or study or studies)).mp,kw.	5656	Advanced
67	(conceal* adj2 allocat*).mp,kw.	3420	Advanced
68	"double blind*".mp,kw.	245920	Advanced
69	"single blind*".mp,kw.	44691	Advanced
70	"triple blind".mp,kw.	822	Advanced
71	"single masked*".mp,kw.	435	Advanced
72	"double masked".mp,kw.	3667	Advanced
73	"triple masked".mp,kw.	73	Advanced
74	placebo*.mp,kw.	438327	Advanced
75	or/49-74	2104162	Advanced
76	48 and 75	1482	Advanced
77	limit 48 to (meta analysis or "systematic review" or randomized controlled trial)	555	Advanced
78	76 or 77	1482	Advanced
79	limit 78 to (books or chapter or conference abstract or conference paper or "conference review")	218	Advanced
80	78 not 79	1264	Advanced

D. **Cochrane Central Register of Controlled Trials February 11, 2019**

#	Searches	Results	Type
1	Hypothyroidism/	374	Advanced
2	hypothyr*.mp,kw.	1478	Advanced
3	hypo-thyr*.mp,kw.	5	Advanced
4	(thyroid? adj3 defic*).mp,kw.	62	Advanced
5	(thyroid? adj3 insuffic*).mp,kw.	13	Advanced
6	(thyroid? adj3 failure?).mp,kw.	37	Advanced
7	(thyroid? adj3 low adj3 product*).mp,kw.	0	Advanced
8	(thyroid? adj3 under adj3 product*).mp,kw.	22	Advanced
9	(thyroid? adj3 underactive*).mp,kw.	3	Advanced
10	(tsh adj3 defic*).mp,kw.	12	Advanced

11	or/1-10	1559	Advanced
12	Triiodothyronine/	541	Advanced
13	triiodothyronin*.mp,kw.	878	Advanced
14	triiodo l thyronin*.mp,kw.	4	Advanced
15	triiodo-thyronin*.mp,kw.	4	Advanced
16	levotriiodothyronin*.mp,kw.	4	Advanced
17	liothyronin*.mp,kw.	412	Advanced
18	lio-thyronin*.mp,kw.	0	Advanced
19	liothyron*.mp,kw.	412	Advanced
20	lyothyronin*.mp,kw.	0	Advanced
21	levoliothyronin*.mp,kw.	0	Advanced
22	L-triiod?thyronin*.mp,kw.	28	Advanced
23	L-T3.mp,kw.	31	Advanced
24	LT3.mp,kw.	16	Advanced
25	[6138-47-2.rn.]	0	Advanced
26	[6893-02-3.rn.]	0	Advanced
27	or/12-26	1138	Advanced
28	Thyroxine/	852	Advanced
29	levothyrox*.mp,kw.	596	Advanced
30	laevothyrox*.mp,kw.	1	Advanced
31	levo-thyrox*.mp,kw.	13	Advanced
32	laevo-thyrox*.mp,kw.	0	Advanced
33	l-thyrox*.mp,kw.	197	Advanced
34	levo-t.mp,kw.	1	Advanced
35	oroxin*.mp,kw.	0	Advanced
36	thyroxin*.mp,kw.	1739	Advanced
37	L-T4.mp,kw.	126	Advanced
38	LT4.mp,kw.	91	Advanced
39	[51-48-9.rn.]	0	Advanced
40	or/28-39	1991	Advanced
41	27 or 40	2299	Advanced
42	11 and 41	721	Advanced
43	limit 42 to (conference or consensus development conference)	20	Advanced
44	42 not 43	701	Advanced

E. **Cochrane Database of Systematic Reviews February 11, 2019**

#	Searches	Results	Type
1	hypothyr*.mp,kw.	148	Advanced
2	hypo-thyr*.mp,kw.	0	Advanced
3	(thyroid? adj3 defic*).mp,kw.	14	Advanced
4	(thyroid? adj3 insuffic*).mp,kw.	3	Advanced
5	(thyroid? adj3 failure?).mp,kw.	6	Advanced
6	(thyroid? adj3 low adj3 product*).mp,kw.	0	Advanced
7	(thyroid? adj3 under adj3 product*).mp,kw.	0	Advanced
8	(thyroid? adj3 underactive*).mp,kw.	5	Advanced
9	(tsh adj3 defic*).mp,kw.	2	Advanced
10	or/1-9	153	Advanced
11	triiodothyronin*.mp,kw.	26	Advanced
12	triiodo l thyronin*.mp,kw.	0	Advanced
13	triiodo-thyronin*.mp,kw.	0	Advanced
14	levotriiodothyronin*.mp,kw.	0	Advanced
15	liothyronin*.mp,kw.	4	Advanced
16	lio-thyronin*.mp,kw.	0	Advanced
17	liothyron*.mp,kw.	4	Advanced
18	lyothyronin*.mp,kw.	0	Advanced
19	levoliothyronin*.mp,kw.	0	Advanced
20	L-triiod?thyronin*.mp,kw.	2	Advanced
21	L-T3.mp,kw.	0	Advanced
22	LT3.mp,kw.	0	Advanced
23	levothyrox*.mp,kw.	15	Advanced
24	laevothyrox*.mp,kw.	0	Advanced
25	levo-thyrox*.mp,kw.	0	Advanced
26	laevo-thyrox*.mp,kw.	0	Advanced
27	l-thyrox*.mp,kw.	6	Advanced
28	levo-t.mp,kw.	0	Advanced
29	oroxin*.mp,kw.	0	Advanced
30	thyroxin*.mp,kw.	43	Advanced
31	L-T4.mp,kw.	3	Advanced
32	LT4.mp,kw.	3	Advanced
33	or/11-32	50	Advanced
34	10 and 33	34	Advanced
35	limit 34 to protocols	6	Advanced
36	34 not 35	28	Advanced

F. **Ovid Emcare Nursing 1995 to 2019 February 11**

#	Searches	Results	Type
1	hypothyroidism/	8378	Advanced
2	hypothyr*.mp,kw.	10009	Advanced
3	hypo-thyr*.mp,kw.	10	Advanced
4	(thyroid? adj3 defic*).mp,kw.	166	Advanced
5	(thyroid? adj3 insuffic*).mp,kw.	45	Advanced
6	(thyroid? adj3 failure?).mp,kw.	85	Advanced
7	(thyroid? adj3 low adj3 product*).mp,kw.	0	Advanced
8	(thyroid? adj3 under adj3 product*).mp,kw.	0	Advanced
9	(thyroid? adj3 underactive*).mp,kw.	6	Advanced
10	(tsh adj3 defic*).mp,kw.	20	Advanced
11	or/1-10	10154	Advanced
12	liothyronine/	3317	Advanced
13	triiodothyronin*.mp,kw.	1304	Advanced
14	triiodo l thyronin*.mp,kw.	35	Advanced
15	triiodo-thyronin*.mp,kw.	3	Advanced
16	levotriiodothyronin*.mp,kw.	1	Advanced
17	liothyronin*.mp,kw.	3665	Advanced
18	lio-thyronin*.mp,kw.	0	Advanced
19	liothyron*.mp,kw.	3665	Advanced
20	lyothyronin*.mp,kw.	0	Advanced
21	levoliothyronin*.mp,kw.	0	Advanced
22	L-triiod?thyronin*.mp,kw.	8	Advanced
23	L-T3.mp,kw.	10	Advanced
24	LT3.mp,kw.	13	Advanced
25	6138-47-2.rn.	1735	Advanced
26	6893-02-3.rn.	1735	Advanced
27	or/12-26	3826	Advanced
28	thyroxine/	5341	Advanced
29	levothyrox*.mp,kw.	4105	Advanced
30	laevothyrox*.mp,kw.	0	Advanced

31	levo-thyrox*.mp,kw.	11	Advanced
32	laevo-thyrox*.mp,kw.	0	Advanced
33	l-thyrox*.mp,kw.	292	Advanced
34	levo-t.mp,kw.	5	Advanced
35	oroxin*.mp,kw.	9	Advanced
36	thyroxin*.mp,kw.	6402	Advanced
37	L-T4.mp,kw.	92	Advanced
38	LT4.mp,kw.	95	Advanced
39	51-48-9.rn.	2324	Advanced
40	or/28-39	9388	Advanced
41	27 or 40	10378	Advanced
42	11 and 41	4009	Advanced
43	(exp animals/ or exp animal experimentation/ or nonhuman/) not ((exp animals/ or exp animal experimentation/ or nonhuman/) and exp human/)	407842	Advanced
44	42 not 43	3893	Advanced
45	limit 44 to (embryo <first trimester> or infant <to one year> or child <unspecified age> or preschool child <1 to 6 years> or school child <7 to 12 years> or adolescent <13 to 17 years>)	620	Advanced
46	limit 44 to (adult <18 to 64 years> or aged <65+ years>)	1996	Advanced
47	45 not 46	445	Advanced
48	44 not 47	3448	Advanced
49	"systematic review"/	88589	Advanced
50	"systematic review (topic)"/	11192	Advanced
51	exp meta analysis/	49718	Advanced
52	exp "meta analysis (topic)"/	14494	Advanced
53	network meta-analysis/	299	Advanced
54	metaanaly*.mp,kw.	2347	Advanced
55	meta-analy*.mp,kw.	82797	Advanced
56	metanaly*.mp,kw.	85	Advanced

57	(systematic* adj3 (review? or study or studies or overview?)).mp,kw.	112952	Advanced
58	exp "randomized controlled trial (topic)"/	56785	Advanced
59	randomized controlled trial/	162405	Advanced
60	exp randomization/	17283	Advanced
61	placebo/	94368	Advanced
62	double blind procedure/	45235	Advanced
63	single blind procedure/	10419	Advanced
64	random*.mp,kw.	449432	Advanced
65	quasirandom*.mp,kw.	79	Advanced
66	(pragmatic* adj4 (trial? or study or studies)).mp,kw.	2307	Advanced
67	(conceal* adj2 allocat*).mp,kw.	1216	Advanced
68	"double blind*".mp,kw.	57727	Advanced
69	"single blind*".mp,kw.	13241	Advanced
70	"triple blind".mp,kw.	287	Advanced
71	"single masked*".mp,kw.	128	Advanced
72	"double masked".mp,kw.	949	Advanced
73	"triple masked".mp,kw.	25	Advanced
74	placebo*.mp,kw.	104279	Advanced
75	or/49-74	587984	Advanced
76	48 and 75	331	Advanced
77	limit 48 to (meta analysis or "systematic review" or randomized controlled trial)	104	Advanced
78	76 or 77	331	Advanced
79	limit 78 to (books or chapter or conference abstract or conference paper or "conference review")	4	Advanced
80	78 not 79	327	Advanced

G. **PsycINFO 1806 to February Week 1 2019**

#	Searches	Results	Type
1	hypothyroidism/	668	Advanced

2	hypothyr*.mp.	1637	Advanced
3	hypo-thyr*.mp.	2	Advanced
4	(thyroid? adj3 defic*).mp.	144	Advanced
5	(thyroid? adj3 insuffic*).mp.	34	Advanced
6	(thyroid? adj3 failure?).mp.	15	Advanced
7	(thyroid? adj3 low adj3 product*).mp.	0	Advanced
8	(thyroid? adj3 under adj3 product*).mp.	0	Advanced
9	(thyroid? adj3 underactive*).mp.	2	Advanced
10	(tsh adj3 defic*).mp.	7	Advanced
11	or/1-10	1734	Advanced
12	triiodothyronine/	231	Advanced
13	triiodothyronin*.mp.	652	Advanced
14	triiodo l thyronin*.mp.	7	Advanced
15	triiodo-thyronin*.mp.	1	Advanced
16	levotriiodothyronin*.mp.	8	Advanced
17	liothyronin*.mp.	16	Advanced
18	lio-thyronin*.mp.	0	Advanced
19	liothyron*.mp.	16	Advanced
20	lyothyronin*.mp.	0	Advanced
21	levoliothyronin*.mp.	0	Advanced
22	L-triiod?thyronin*.mp.	32	Advanced
23	L-T3.mp.	3	Advanced
24	LT3.mp.	4	Advanced
25	or/12-24	673	Advanced
26	thyroxine/	356	Advanced
27	levothyrox*.mp.	164	Advanced
28	laevothyrox*.mp.	0	Advanced
29	levo-thyrox*.mp.	4	Advanced
30	laevo-thyrox*.mp.	0	Advanced
31	l-thyrox*.mp.	78	Advanced
32	levo-t.mp.	1	Advanced
33	oroxin*.mp.	0	Advanced
34	thyroxin*.mp.	1070	Advanced
35	L-T4.mp.	18	Advanced
36	LT4.mp.	10	Advanced
37	or/26-36	1192	Advanced

38	25 or 37	1526	Advanced
39	11 and 38	417	Advanced
40	limit 39 to animal	125	Advanced
41	limit 39 to human	287	Advanced
42	40 not 41	121	Advanced
43	39 not 42	296	Advanced
44	limit 43 to (100 childhood <birth to age 12 yrs> or 120 neonatal <birth to age 1 mo> or 140 infancy <2 to 23 mo> or 160 preschool age <age 2 to 5 yrs> or 180 school age <age 6 to 12 yrs> or 200 adolescence <age 13 to 17 yrs>)	66	Advanced
45	limit 43 to ("300 adulthood <age 18 yrs and older>" or 320 young adulthood <age 18 to 29 yrs> or 340 thirties <age 30 to 39 yrs> or 360 middle age <age 40 to 64 yrs> or "380 aged <age 65 yrs and older>" or "390 very old <age 85 yrs and older>")	183	Advanced
46	44 not 45	40	Advanced
47	43 not 46	256	Advanced
48	limit 47 to ("0200 book" or "0240 authored book" or "0280 edited book" or "0300 encyclopedia" or "0400 dissertation abstract" or chapter or dissertation or review-book or review-media or review-software & other)	8	Advanced
49	47 not 48	248	Advanced

H. **Web of Science from** Clarivate Analytics **February 11, 2019**

6

866
#4 AND #3
Refined by: DOCUMENT TYPES: (ARTICLE) AND [excluding] DOCUMENT TYPES: (PROCEEDINGS PAPER)
Indexes=SCI-EXPANDED, SSCI, A&HCI, ESCI Timespan=All years

5

892
#4 AND #3
Refined by: DOCUMENT TYPES: (ARTICLE)
Indexes=SCI-EXPANDED, SSCI, A&HCI, ESCI Timespan=All years

4

1,656,808
(TS=((systematic* W/3 (review? or overview? or study or studies)) OR meta-anal* OR metaanal* OR metanal* OR random* OR quasirandom* OR (pragmatic* W/3 (trial? or study or studies)) OR "single blind*" OR "double blind*" OR "triple-blind*" OR "single masked*" OR "double masked" OR "triple masked" OR placebo*)) AND DOCUMENT TYPES: (Article)
Indexes=SCI-EXPANDED, SSCI, A&HCI, ESCI Timespan=All years

3

8,659
#2 AND #1
Indexes=SCI-EXPANDED, SSCI, A&HCI, ESCI Timespan=All years

2

32,550
(TS=(triiodothyronin* OR triiodo l thyronin* OR triiodo-thyronin* OR levotriiodothyronin* OR liothyronin* OR lio-thyronin* OR liothyron* OR lyothyronin* OR levoliothyronin* OR L-triiod?thyronin* OR L-T3 OR LT3 OR levothyrox* OR laevothyrox* OR levo-thyrox* OR laevo-thyrox* OR l-thyrox* OR levo-t OR oroxin* OR thyroxin* OR L-T4 OR LT4)) AND DOCUMENT TYPES: (Article)
Indexes=SCI-EXPANDED, SSCI, A&HCI, ESCI Timespan=All years

1

25,487
(TS=(hypothyr* OR hypo-thyr* OR (thyroid? W/3 defic*) OR (thyroid? W/3 insuffic*) OR (thyroid? W/3 failure?) OR (thyroid? W/3 low W/3 product*) OR (thyroid? W/3 under W/3 product*) OR (thyroid? W/3 underactive*) OR (tsh W/3 defic*))) AND DOCUMENT TYPES: (Article)
Indexes=SCI-EXPANDED, SSCI, A&HCI, ESCI Timespan=A

I. **ClinicalTrials.gov February 12, 2019**

#1 (hypothyroid OR hypothyroidism)

#2 (triiodothyronin* OR liothyronin* OR thyroxin* OR levothyrox* OR L-T3 OR LT3 OR L-T4 OR LT4) |

#1 AND #2 89 Trials

Supplementary Table 1. Reviewed full-text papers that were excluded (with reason)

**Clinical practice guideline, with no new original study data**	Biondi B, Bartalena L, Chiovato L, Lenzi A, Mariotti S, Pacini F, et al. (2016). Recommendations for treatment of hypothyroidism with levothyroxine and levotriiodothyronine: A 2016 position statement of the Italian Society of Endocrinology and the Italian Thyroid Association. *J Endocrinol Invest*. (2016) 39:12, 1465-1474. 10.1007/s40618-016-0511-z
	Brenta G, Vaisman M, Sgarbi JA, Bergoglio LM, Andrada NC, Bravo, P. P., et al. Clinical practice guidelines for the management of hypothyroidism. *Arq Bras Endocrinol Metabol*. (2013) 57:4, 265-91.
	Dave JA, Klisiewicz A, Bayat Z, Mohamed NA, Stevens, Z, Mollentze WF, et al. SEMDSA/ACE-SA guideline for the management of hypothyroidism in adults. *J Endocrinol, Metab and Diab of S Afr*. (2015) 20:2, 18-26.
	Garber JR, Cobin RH, Gharib H, Hennessey JV, Klein I, Mechanick JI, et al. Clinical Practice Guidelines for Hypothyroidism in Adults: the American Association of Clinical Endocrinologists and the American Thyroid Association. *Thyroid.* (2012) 22:12, 1200-1235. 10.1089/thy.2012.0205
	Guglielmi R, Frasoldati A, Zini M, Grimaldi F, Gharib H, Garber JR, et al. Italian Association of Clinical Endocrinologists statement – Replacement therapy for primary hypothyroidism: A brief guide for clinical practice. *Endocr Pract.* (2016) 22:11, 1319-1326. 10.4158/ep161308.or
	Jonklaas J, Bianco AC, Bauer AJ, Burman KD, Cappola AR, Celi FS, et al. Guidelines for the Treatment of Hypothyroidism: Prepared by the American Thyroid Association Task Force on Thyroid Hormone Replacement. *Thyroid.* (2014) 24:12, 1670-1751. 10.1089/thy.2014.0028

	Okosieme O, Gilbert J, Abraham P, Boelaert K, Dayan C, Gurnell M, et al. Management of primary hypothyroidism: Statement by the British Thyroid Association Executive Committee. *Clin Endocrinol*. (2016) 84:6, 799-808. http://dx.doi.org/10.1111/cen.12824
**Narrative review, editorial or commentary with no new original data**	Biondi B, Wartofsky L. Combination treatment with T4 and T3: Toward personalized replacement therapy in hypothyroidism? *J Clin Endocrinol Metab*. (2012) 97:7, 2256-2271. http://dx.doi.org/10.1210/jc.2011-3399
	Escobar-Morreale HFF, Botella-Carretero JI, De Escobar GM. Treatment of hypothyroidism with levothyroxine or a combination of levothyroxine plus L-triiodothyronine. *Best Pract Res Clin Endocrinol Metab*. (2015) 29:1, 57-75. http://dx.doi.org/10.1016/j.beem.2014.10.004
	Hennessey JV, Espaillat R. Current evidence for the treatment of hypothyroidism with levothyroxine/levotriiodothyronine combination therapy versus levothyroxine monotherapy. *Int J Clin Pract*. (2018) 72:2. https://dx.doi.org/10.1111/ijcp.13062
	Hoermann R, Midgley JEM, Larisch R, Dietrich JW. Lessons from randomised clinical trials for triiodothyronine treatment of hypothyroidism: Have they achieved their objectives? *J Thyroid Res*. (2018) 2018:3239197. http://dx.doi.org/10.1155/2018/3239197
	Jonklaas J. Risks and safety of combination therapy for hypothyroidism. Expert Rev *Clin Pharmacol*. (2016) 9:8, 1057-67. https://dx.doi.org/10.1080/17512433.2016.1182019
	Linder LM, Clements JN. Is combination therapy appropriate for hypothyroidism? *J Am Acad Physician Assist*. (2015) 28:9, 16-19. https://dx.doi.org/10.1097/01.jaa.0000470438.14941.98

	Ma C, Xie J, Huang X, Wang G, Wang Y, Wang X, et al. Thyroxine alone or thyroxine plus triiodothyronine replacement therapy for hypothyroidism. *Nucl Med Commun*. (2009) 30:8, 586-93. https://dx.doi.org/10.1097/MNM.0b013e32832c79e0
	McDermott MT. Does combination T4 and T3 therapy make sense?. *Endocr Pract*. (2012) 18:5, 750-7. https://dx.doi.org/10.4158/EP12076.RA
	Pinchera A, Santini F. Is combined therapy with levothyroxine and liothyronine effective in patients with primary hypothyroidism?. *Nat Clin Pract Endocrinol Metab*. (2005) 1:1, 43726.
	Wiersinga WM. L-T4 and L-T3 combined treatment vs L-T4 alone. *Ann Endocrinol (Paris)*. (2007) 68:4, 216-9.
	Wiersinga WM. Do we need still more trials on T4 and T3 combination therapy in hypothyroidism?. *Eur J Endocrinol*. (2009) 161:6, 955-9. https://dx.doi.org/10.1530/EJE-09-0879
	Wiersinga WM. Therapy of endocrine disease: T4+T3 combination therapy: is there a true effect?. *Eur J Endocrinol*. (2017) 177:6, R287-R296. https://dx.doi.org/10.1530/EJE-17-0645
**No combination therapy preference outcome data**	Bunevicius R, Prange Jr AJ. Mental improvement after replacement therapy with thyroxine plus triiodothyronine: Relationship to cause of hypothyroidism. *Int J Neuropsychopharmacol*. (2000) 3:2, 167-174. http://dx.doi.org/10.1017/S1461145700001826
	Clyde PW, Harari AE, Getka EJ, Shakir KMM. Combined levothyroxine plus liothyronine compared with levothyroxine alone in primary hypothyroidism - A randomized controlled trial. *JAMA*. (2003) 290:22, 2952-2958. 10.1001/jama.290.22.2952

Joffe RT, Sawka AM, Marriott MJ, MacQueen GM, Gernstein HC. Does substitution of T4 with T3 plus T4 for T4 replacement improve depressive symptoms in patients with hypothyroidism?. *Ann N Y Acad Sci*. (2004) 1032, 287-8.

Kaminski J, Miasaki FY, Paz-Filho G, Graf H, Carvalho GA. Treatment of hypothyroidism with levothyroxine plus liothyronine: a randomized, double-blind, crossover study. *Arch Endocrinol Metab*. (2016) 60:6, 562-572. https://dx.doi.org/10.1590/2359-3997000000192

Krysiak R, Szkrobka W, Okopien B. Sexual function and depressive symptoms in young women with hypothyroidism receiving levothyroxine/liothyronine combination therapy: a pilot study. *Curr Med Res Opin*. (2018) 34:9, 1579-1586. http://dx.doi.org/10.1080/03007995.2018.1448771

Panicker V, Saravanan P, Vaidya B, Evans J, Hattersley AT, Frayling TM, et al. Common Variation in the DIO2 Gene Predicts Baseline Psychological Well-Being and Response to Combination Thyroxine Plus Triiodothyronine Therapy in Hypothyroid Patients. *J Clin Endocrinol Metab*. (2009) 94:5, 1623-1629. 10.1210/jc.2008-1301

Paz-Filho G, Kaminski J, Miasaki FY, De Carvalho GA.Treatment of hypothyroidism with levothyroxine plus triiodothyronine: A randomized, double-blind crossover study. *Thyroid*. (2015) 25, A4.

Saravanan P, Simmons DJ, Greenwood R, Peters TJ, Dayan CM. Partial substitution of thyroxine with tri-iodothyronine in patients on T4 replacement therapy: Results of a large community-based randomized controlled trial. *J Clin Endocrinol Metab*. (2005) 90:2, 805-812. http://dx.doi.org/10.1210/jc.2004-1672

Saravanan P, Visser TJ, Dayan CM. Psychological well-being correlates with free thyroxine but not free 3,5,3'-

	triiodothyronine levels in patients on thyroid hormone replacement. *J Clin Endocrinol Metab*. (2006) 91:9, 3389-93.
	Sawka AM, Gerstein HC, Marriott MJ, MacQueen GM, Joffe RT. Does a Combination Regimen of Thyroxine and 3,5,3'-Triiodothyronine Improve Depressive Symptoms Better Than T4 Alone in Patients with Hypothyroidism? Results of a Double-Blind, Randomized, Controlled Trial. *J Clin Endocrinol Metab*. (2003) 88:10, 4551-4555. http://dx.doi.org/10.1210/jc.2003-030139
	Schmidt U, Nygaard B, Jensen EW, Kvetny J, Jarlov A, Faber J. Peripheral markers of thyroid function: The effect of T4 monotherapy vs T4/T3 combination therapy in hypothyroid subjects in a randomized crossover study. *Endocr Connect*. (2013) 2:1, 55-60. http://dx.doi.org/10.1530/EC-12-0064
	Siegmund W, Spieker K, Weike AI, Giessmann T, Modess C, Daber, T, et al. Replacement therapy with levothyroxine plus triiodothyronine (bioavailable molar ratio 14 : 1) is not superior to thyroxine alone to improve well-being and cognitive performance in hypothyroidism. *Clin Endocrinol*. (2004) 60:6, 750-7.
	Valizadeh M, Seyyedmajidi MR, Momtazi S, Musavi N. The efficacy of combined levothyroxine plus liothyronine with levothyroxine alone in primary hypothyroidism: a randomized controlled trial. Iran J Endocrinol Metab. (2009) 10:5, 465.
	Valizadeh M, Seyyed-Majidi MR, Hajibeigloo H, Momtazi S, Musavinasab N, Hayatbakhsh MR.. Efficacy of combined levothyroxine and liothyronine as compared with levothyroxine monotherapy in primary hypothyroidism: a randomized controlled trial. *Endocr Res*. (2009) 34:3, 80-9. https://dx.doi.org/10.1080/07435800903156340

**Observational study (not randomized) on T3/T4 combination therapy**	Michaelsson LF, La Cour JL, Medici BB, Watt T, Faber J, Nygaard B. Levothyroxine/Liothyronine Combination Therapy and Quality of Life: Is It All about Weight Loss?. *Eur Thyroid J*. (2018) 7:5, 243-250. 10.1159/000490383
	Tariq A, Wert Y, Cheriyath P, Joshi R. Effects of Long-Term Combination LT4 and LT3 Therapy for Improving Hypothyroidism and Overall Quality of Life. *South Med J*. (2018) 111:6, 363-369. 10.14423/smj.0000000000000823
	Regalbuto C, Maiorana R, Alagona C, Di Paola R, Cianci M, Alagona G, et al. Effects of either LT4 monotherapy or LT4/LT3 combined therapy in patients totally thyroidectomized for thyroid cancer. *Thyroid*. (2007) 17:4, 323-331. http://dx.doi.org/10.1089/thy.2006.0084
**Manuscript not in English**	Nygaard B, Roder E, Karmisholt J, Kristensen JK. Combination therapy of thyroxine and triiodothyronine in hypothyroid patients. *Ugeskrift for laeger*. (2018) 180:19.
	Tellez TR. Critical appraisal: combined T3 and T4 replacement therapy is not better than replacement with T4 alone. *Rev Med Chil*. (2005) 133:9, 1116-9.
**Unblinded randomized-controlled trial**	Fadeyev VV, Morgunova TB, Melnichenko GA, Dedov II. Combined therapy with L-Thyroxine and L-Tiiodothyronine compared to L-Thyroxine alone in the treatment of primary hypothyroidism. *Hormones*. (2010) 9:3, 245-252.
	Fadeyev VV, Morgunova TB, Sytch JP, Melnichenko GA. TSH and thyroid hormones concentrations in patients with hypothyroidism receiving replacement therapy with L-thyroxine alone or in combination with L-triiodothyronine. *Hormones*. (2005) 4:2, 101-7.

**No TSH measurement in patients in the study**	Smith KM. Controlled clinical trial of combined triiodothyronine and thyroxine in the treatment of hypothyroidism. *Br Med J* (1970). 4:5728, 145-148.
**No use of synthetic T3T4 combination therapy**	Walsh JP, Ward LC, Burke V, Bhagat CI, Shiels L, Henley D., et al. Small changes in thyroxine dosage do not produce measurable changes in hypothyroid symptoms, well-being, or quality of life: results of a double-blind, randomized clinical trial. *J Clin Endocrinol Metab*. (2006) 91:7, 2624-30.
**Systematic review on this topic**	Escobar-Morreale HF, Botella-Carretero JI, Escobar del Rey F, Morreale de Escobar G. Review: Treatment of hypothyroidism with combinations of levothyroxine plus liothyronine. *J Clin Endocrinol Metab*. (2005) 90:8, 4946-54.
	Fischman A, Dominguez JM. Combined therapy with levothyroxine and liothyronine for hypothyroidism. *Medwave*. (2018) 18:8, e7376. http://dx.doi.org/10.5867/medwave.2018.08.7375
	Grozinsky-Glasberg S, Fraser A, Nahshoni E, Weizman A, Leibovici L. Thyroxine-triiodothyronine combination therapy versus thyroxine monotherapy for clinical hypothyroidism: meta-analysis of randomized controlled trials. *J Clin Endocrinol Metab*. (2006) 91:7, 2592-9.
	Joffe RT, Brimacombe M, Levitt AJ, Stagnaro-Green A. Treatment of clinical hypothyroidism with thyroxine and triiodothyronine: a literature review and metaanalysis. *Psychosomatics*. (2007) 48:5, 379-84.
**Systematic review of clinical practice guidelines**	Kraut E, Farahani P. A Systematic review of clinical practice guidelines' recommendations on levothyroxine therapy alone versus combination therapy (LT4 plus LT3) for hypothyroidism. *Clin Invest Med*. (2015) 38:6, E305-13.

**Study protocol document**	Combined levothyroxine/liothyronine supplementation in hypothyroid patients with brain tumors. ClinicalTrials.gov Identifier: NCT00488644
	Effect of T4-T3 combination therapy versus T4 monotherapy in patients with hypothyroidism. ClinicalTrials.gov Identifier: NCT00531713<br><br>(published trial by Nygaard et al., reference 18)
	Desiccated thyroid extract versus synthetic T3/T4 combination (ThyrolarTM) versus L-T4 alone in the therapy of primary hypothyroidism with special attention to the gene polymorphism. ClinicalTrials.gov Identifier: NCT02317926.
	Combined replacement therapy with levothyroxine and liothyronine in thyroidectomized patients. Combined replacement therapy with levothyroxine and liothyronine in thyroidectomized patients: Effects on peripheral tissues. A prospective, randomized, controlled, double-blind study. ClinicalTrials.gov Identifier: NCT03053115.
	Combined therapy with L-Thyroxine and L-Triiodothyronine compared to L-thyroxine alone. ClinicalTrials.gov Identifier: NCT00715572 .<br><br>(Published trial by Fadayev et al. [reference 30]).
